# Economic situation of informal caregivers for older adults in the context of changing long-term care policies: a mixed methods study

**DOI:** 10.1186/s12889-026-28179-2

**Published:** 2026-06-22

**Authors:** Motoko Ishikawa, Laura Saarukka, Minna Zechner

**Affiliations:** Faculty of Social Sciences, University of Helsinki, (Unioninkatu 37), P.O. Box 54, Helsinki, 00014 Finland

**Keywords:** Cash-for-care schemes, Informal Care Allowance, Finland, Informal caregivers, Economic situation, Costs of care

## Abstract

**Background:**

Long-term care policies across Europe increasingly rely on informal care amid population ageing and constraints in public expenses. Cash-for-care schemes have been introduced to support informal care and alleviate socio-economic differences between caregivers. This mixed methods study investigates the subjective economic experiences of informal caregivers for older adults in Finland, where the policy initiative has gradually shifted the emphasis of care services from public provision to informal care with the support of Informal Care Allowance (ICA).

**Methods:**

Drawing from a nationwide survey targeting informal caregivers, we focused on respondents who provided care for older adults aged 65 and over and answered the question on their economic situation (*N* = 1057). The study employed logistic regression analysis and qualitative content analysis to examine factors associated with economic hardship and caregivers’ lived experiences.

**Results:**

Quantitative findings revealed that younger caregivers, those with poor health, those caring for someone other than their spouse, caregivers providing various types of care, those lacking public sector support, those receiving adequate support from the church social work or having unmet church social work-related needs, non-recipients of informal care allowance, and individuals forced to leave employment faced significantly weakened economic situations. Qualitative analysis identified six themes on the economic impact of caregiving: dissatisfaction with the informal care allowance, utility of the informal care allowance, increase in care costs, adverse economic consequences, managing costs associated with care, and caregiving at the sacrifice of working life.

**Conclusions:**

Informal caregivers in Finland, especially those with low incomes or reduced work due to caregiving, are facing significant financial strain. This may reflect broader shifts in long-term care policy and a decreased public responsibility. While the informal care allowance can help improve caregivers’ situations, it remains insufficient, particularly for working-age individuals whose earnings and career opportunities are affected. This could worsen social inequalities in both the short and long term. The divergent interpretations of the purpose of the ICA system underscore the scheme’s inadequacy in meeting the diverse needs of caregivers. Future policies should better account for varying circumstances, providing stronger financial support and enabling caregivers to balance employment with care responsibilities.

**Supplementary Information:**

The online version contains supplementary material available at https://doi.org/10.1186/s12889-026-28179-2.

## Introduction

Long-term care of older adults is a burning policy issue, with predominant focus on public expenses [1]. As the demand for long-term care is increasing with population ageing, governments tend to place their hope on informal care, provided by families, kin and friends whereas formal care refers to care from public and private services. It has been estimated that from 2000 to 2050 the burden of informal care for older adults is projected to increase by 49.7% [2].

The question of informal care is not only about addressing the care needs of older adults. It is very much a question of equality, as socioeconomically disadvantaged groups, referring to lower levels of income, wealth and education, are providing the bulk of informal care [3]. If those providing informal care are already in a more economically disadvantaged situation, placing further emphasis on informal care will exacerbate socio-economic differences within those giving as well as those in need of care.

One way to alleviate these socio-economic differences and support informal care is through cash-for-care schemes, which have been essential components of long-term care (LTC) policies across Europe since the 1990s. These schemes involve publicly funded monetary transfers provided to older adults in need of care to compensate informal caregivers or purchase professional or other market services [4, 5]. From the users’ point of view, receiving cash benefits instead of services in kind is a fundamental feature of the policy.

Focusing on cash for those in need of care is a rather narrow definition of these schemes and excludes cash benefits provided directly to caregivers with the aim of supporting and fostering informal caregiving. Broader understandings of cash-for-care schemes, however, encompass different models in which cash benefits are paid directly to carers to compensate for the loss of earned income or direct payments are provided to informal carers to replace publicly funded formal care [6]. Regardless of the differences in scope and the way of provision, the common objectives of cash-for-care schemes are ensuring choice for users, developing care markets, recognizing and fostering family care, facilitating deinstitutionalization, and containing LTC costs [7, 8]. At the same time, cash-for-care diminishes the public responsibility over the supply and quality of care and pulls especially middle-aged women out of the labor market. This, in turn, negatively affects the income levels of caregivers as the benefit levels are generally not high enough to ensure proper income [9–11].

It is thus evident that LTC policies play a central role in affecting the economic situation of present and future informal caregivers of older adults. As LTC policies are in flux due to changes in demography, austerity politics, and an increasing emphasis on ageing in place [12, 13], it is important to pay attention to the economic consequences of informal caregiving from the caregivers’ perspective. This study aims to investigate the hitherto less studied subjective views of informal caregivers [see, however, 14–16] toward their economic situation by applying mixed methods. The context is Finland, where the public provision of care services has gradually shifted to rely more on informal care [11, 13, 17] with and without the support of cash-for-care, namely the Informal Care Allowance.

## Background

### Cash-for-care schemes in the context of long-term care

Informal Care Allowance (ICA) in Finland is a cash-for-care scheme in a broader sense paid to caregivers. It includes an allowance for the caregiver, services for the care receiver, carer’s days off with substitute care, support services, insurance, and health check-ups and training if needed for the caregiver. The Act on Support for Informal Care (937/2005) [18] defines the conditions for granting ICA and its contents, including the minimum allowance level of 530 € per month in 2026. The level of allowance is determined by the binding and demanding nature of care, but there are no national regulations, meaning that allowance levels for similar caregiving situations may vary across the country. The goal is to enhance informal care for the benefit of care receivers by securing sufficient social and health services, continuity of caregiving, and support for informal caregivers [19]. ICA is not a subjective right but is discretionarily applied by the wellbeing services county where the care receiver resides. The coverage of ICA for the care of older adults aged 75 and over is around 4% of the age group and has been decreasing since 2018 [20]. The decrease means that while the eligibility criteria for formal care services, both domiciliary and institutional, have been tightening, ICA is less available as well [17]. As a result, more informal care is likely being provided without public support. The importance of informal care is highlighted in the Government Program, stating that “In an ageing society, the contribution of informal care is increasing, and the Government considers informal care a valuable part of a Finnish welfare service” [21].

Finland’s broader economic environment is characterized by a combination of high labour market participation, a comprehensive and earnings-related pension system, and several pathways that support gradual retirement or continued work later in life. In 2023, the employment rate of the working age population (15–64 years old) was 74%, 3.6% points higher than the EU27 average, with both women (74.1%) and men (73.9%) equally participating in the labor market. In contrast, the unemployment rate is consistently above the EU27 average (7.3% vs. 6.1% in 2023) [22]. Finland’s pension system consists of earnings-related pensions based on employment income and national and guarantee pensions based on residence, targeting those with small or no earnings-related pension. The system includes old age, disability, survivors’, and partial old age pensions, ensuring income in old age, helping individuals remain employed even with reduced work ability, and allowing older workers to reduce working hours or transition gradually into retirement [23]. Unemployment security is also comprehensive, consisting of basic unemployment allowance, earnings-related unemployment allowance, and labour market subsidy [24].

Compared to the general economic and labour market status of the entire population in Finland, informal caregivers are in a more vulnerable situation. Out of the 46,553 caregivers receiving ICA in 2025, half were of working age (15–64 years old), while the other half were 65 years old or older. However, only 32% of all caregivers with ICA were employed, 53% were on pension, and 15% were either unemployed, students, or outside the labour market. The most common amount of the allowance was 400–599 euros per month, received by 56% of the caregivers. 70% of informal caregivers were women, while 30% were men [25].

Similar political and practical emphasis on informal care was visible across Europe in a comparative study on the transformation of cash-for-care schemes from the early 1990s to the mid-2010s in seven countries (Austria, England, France, Germany, Italy, the Netherland, and Spain) representing different welfare regimes [5]. It was concluded that despite considerable difference in original goals and policy designs, increasing care needs and cost containment efforts have converted cash-for-care schemes into essential pillars of LTC policies that aim to foster informal care, low-paid care, or both [4, 5]. This trend is relevant to the risk of increasing inequalities within citizens and especially disadvantaging low-income individuals and families [11].

### Economic costs of informal caregiving

While theoretical studies on cash-for-care schemes utilizing predominantly policy documents have been accumulated [4, 5] and the economic consequences of informal caregiving have been studied [14, 15, 26], there is a paucity of empirical research that explores the economic experiences of informal caregivers under the transforming political landscape of long-term care.

Some review studies have synthesized the general knowledge of economic costs incurred by informal caregivers to older adults with care needs. Keating et al. [27] identified three broad domains of sources of economic costs for family members caring for adults with long term health problems and disabilities: employment consequences, out-of-pocket expenses, and caregiving labor. These cost domains respectively have their economic outcomes such as lost income and benefits, reduced savings and ability to meet expenses, and lost economic value of time spent in care labor. Another systematic review found that socio-demographic characteristics of caregivers and care receivers, duration of illness, household income, the number of illnesses, and duration of the care given constitute the multifaceted factors contributing to financial strain among caregivers [28].

A Canadian study found that nearly 18% of caregivers who reported out-of-pocket expenditures experienced financial hardship due to caregiving. The most common expenditure was caused by transportation, travel, and accommodation, medication was the second, and home modifications and assistive devices also created substantial costs for caregivers [29]. A review on costs to caregivers identified that caregivers providing hands-on care, palliative care, or caring for individuals with demanding needs such as dementia or intellectual disabilities experienced high economic costs. Male caregivers also reported higher levels of costs compared to women [30].

## Methods

### Research questions

While informal care may seem like a cost-effective solution for policy makers to address increasing demands for long-term care, the costs of care are actually borne by individuals providing and receiving care. Therefore, this study focuses on the subjective views of caregivers on their economic situation. It aims to address the following questions:

1. What socio-demographic and situational factors are associated with a weakened economic situation among informal caregivers due to providing care for older adults?
2. What is the association between receiving ICA and caregivers’ economic situation after adjusting for other factors?
3. How do informal caregivers experience the impacts of caregiving on their economic situation?

### Data

This study utilized a dataset from a nationwide survey that explored support needs among informal caregivers and care receivers, the use of services and service experiences, and the impacts of caregiving on carers’ subjective health and economic situation. Carers Finland, an advocacy and support association for informal caregivers, conducted the online survey between March and May 2024 [31]. The survey targeted family members who regularly provide care for persons with sickness or disability, and older relatives with physical or cognitive impairments. It also attempted to reach caregivers without receiving ICA. The questionnaire was distributed on the social media channels of Carers Finland and via member associations, as well as forwarded by email to collaborative stakeholders. The questionnaire included multiple-choice and open-ended questions in which respondents described their answers in a free form. The survey received 1,929 respondents across all wellbeing services counties in Finland. This study only included respondents who provided care for older adults aged 65 and over and answered the question on their economic situation, resulting in 1,057 respondents in our final sample.

The survey did not require ethical approval from an ethics committee as it did not involve the collection of personal data or processing sensitive information in accordance with the EU General Data Protection Regulation (GDPR) (2016/679). However, the survey complied with the GDPR and the Finnish Data Protection Act (1050/2018), and this study strictly followed the ethical guidelines established by the Finnish National Board on Research Integrity TENK. Participants were informed through a written privacy notice provided by Carers Finland that participation in the survey was voluntary and that all collected data would be kept confidential and anonymized. Returning the completed questionnaire was considered as providing informed consent to participate in the study.

### Mixed methods approach

Quantitative and qualitative questions included in the survey made it possible to employ a mixed methods approach to address research questions. When clarifying an appropriate research design, we identified two rationales for conducting a mixed methods study: triangulation and illustration of data. Triangulation allows for greater validity in a study by seeking corroboration between quantitative and qualitative data, while a qualitative approach illustrates quantitative findings and helps to paint a more nuanced picture of the phenomenon under investigation [32]. Among the typology of mixed methods designs, a convergent parallel design was selected because the simultaneous collection of quantitative and qualitative data enables the triangulation of findings and enhances the validity of the results [33]. In applying this design, the quantitative and qualitative phases occur simultaneously, both methods are given equal weight, results of each analysis are presented separately, and integration of methods occurs when interpreting the findings.

### Analyzing quantitative data

The quantitative part of the study addressed the research questions (1) and (2), which involved identifying factors associated with a weakened economic situation among informal caregivers and examining the significant role of the ICA after adjusting for other factors. The economic impact of caregiving was assessed in the survey by asking participants: “How has informal caregiving affected your economic situation?” This question served as the dependent variable. Since the study focuses on examining the negative economic effects of caregiving, the four-scale response options were dichotomized into *negative economic effect* (“economic situation weakened”) and *non-negative economic effect* (“not any impact”, “economic situation improved”, and “cannot say”). The response option “cannot say” was grouped with the non-negative economic effect category as it did not convey a negative overtone. Due to the relatively large number of responses to the “cannot say” option (*n* = 183), the responses were retained rather than being considered missing data. The independent variables included caregiver age, care receiver age, caregiver gender, care receiver gender, caregiver’s educational level, caregiver’s subjective health, receipt of Informal Care Allowance (ICA), number of people caring for, to whom care is provided, household structure, duration of care, sum of care types, intensity of caregiving, support received from various sources, use of private paid services, social and health service needs of the caregiver, and leaving employment due to caregiving.

The analysis began with reporting descriptive statistics of dependent and independent variables. Logistic regression models were then conducted to examine the association of various caregiving characteristics with the negative economic impact on the caregiver. Initially, separate logistic models were conducted for each independent variable to estimate their unadjusted associations with the dependent variable (Model 1). Subsequently, a multivariable logistic regression model was fitted, including all independent variables simultaneously, to examine their associations with the dependent variable after adjustment for the other covariates (Model 2). To test whether the main conclusions hold up when the response options for the dependent variables are treated differently, we conducted additional analyses as a robustness check. In the first analysis, the “cannot say” responses were excluded from the binary model. The second analysis treated these responses as a separate outcome category using a three-category multinomial logistic regression model: negative economic effect, cannot say, and non-negative economic effect.

Additionally, Model 1 was performed separately based on the gender of caregivers. This was followed by analyzing interactions between independent variables and caregiver gender using the pooled data to identify whether the associations of individual factors with a weakened economic situation varied between male and female caregivers. Then, tests of interaction terms (each independent variable x caregiver gender) were performed to examine the statistical significance of the difference between the genders. The results of the logistic regression models were presented in odds ratios (OR) with 95% confidence intervals (CI). The models were estimated using Stata/MP 19.0 for Windows statistical software.

### Analyzing qualitative data

The qualitative part of the study addressed the research question (3) by exploring responses to an open-ended question about how caregiving has influenced respondents’ economic situation. The following question in the survey asked participants to describe their situation in a spontaneous manner: “Here, if you wish, you can provide more detailed information about the impact of care on your household economy.” The question received 356 responses in total: 176 responses from those who care for persons aged between 65 and 79, and 180 responses from those who care for older adults aged 80+.

Content analysis was employed to analyze these responses, which varied in length from a few words to long descriptions. The unit of analysis for the qualitative data was a phrase or sentence that conveyed meaning. However, a group of sentences was selected as an analytical unit when one sentence did not capture a meaning sufficiently. Both manifest and latent content were analyzed inductively, following the methodological guidance on open coding, grouping, creating categories, and abstraction [34, 35].

## Findings from the quantitative study

### Sample characteristics

Table 1 displays characteristics of the total sample according to categorized groups of the dependent variable and independent variables. 28% of the respondents reported that their economic situation had weakened due to informal caregiving, while 12% experienced improvement. 72% of the respondents received informal care allowance. While many of the caregivers were older than working age (65 + years old), care receivers were relatively evenly distributed between those aged 65–79 years and those aged 80 years or older. Gender distribution in caregivers and care receivers showed a contrast: females dominated as caregivers, whereas more than 60% of care receivers were male. Most caregivers had completed intermediate education, which refers to graduating from upper secondary education or vocational education. Those who subjectively perceived their health as poor exceeded one tenth of the caregivers. Most of the respondents cared for one family member who was likely to be their spouse. Living with the care receiver was the dominant household structure. The duration of care varied, but over 90% were engaged in caregiving for longer than one year. Care and support offered encompassed a spectrum ranging from instrumental, informational and emotional support to personal care and hygiene. Caregiving was generally intense, with 40% of the respondents involved in continuous, round the clock caregiving. To maintain informal care, caregivers received and required support from various sources. 84% of the respondents needed support from public sector social and health care professionals, and 26% used private paid services. One fifth of the respondents needed to use social and health services for themselves to cope with caregiving duties. Although being a minority, about 6% had to leave their employment due to caregiving.

**Table 1:** Sample characteristics

Variable	*N* (%)
Impact of informal caregiving on caregiver’s economic situation	1,057 (100.0)
Economic situation weakened	295 (27.9)
Not any impact	458 (43.3)
Cannot say	183 (17.3)
Economic situation improved	121 (11.5)
Receipt of informal care allowance	1,036 (100.0)
Yes	745 (71.9)
No	291 (28.1)
Caregiver age	1,046 (100.0)
Working age (< 65 years old)	259 (24.8)
Older than working age (65 + years old)	787 (75.2)
Care receiver age	1,046 (100.0)
65–79 years old	592 (56.6)
80 + years old	454 (43.4)
Caregiver gender	1,048 (100.0)
Male	220 (21.0)
Female	828 (79.0)
Care receiver gender	1046 (100.0)
Male	648 (62.0)
Female	398 (38.0)
Caregiver’s educational level	1,048 (100.0)
Primary education	127 (12.1)
Intermediate education (upper secondary or vocational education)	606 (57.8)
Higher education (either a bachelor’s or master’s degree)	315 (30.1)
Caregiver’s subjective health	1048 (100.0)
Good or relatively good	440 (42.0)
Moderate	471 (44.9)
Quite poor or poor	137 (13.1)
Number of people caring for	1,048 (100.0)
1 person	947 (90.4)
>1 person	101 (9.6)
Giving care to	1,048 (100.0)
Own spouse	740 (70.6)
Other than spouse	308 (29.4)
Living in the same household as the care receiver	1,048 (100.0)
Yes	866 (82.6)
No	182 (17.4)
Duration of care	1,048 (100.0)
Less than a year	76 (7.3)
1–4 years	516 (49.2)
5–10 years	312 (29.8)
More than 10 years	140 (13.4)
Non-response	4 (0.4)
Care types forming a sum variable	
Personal care and hygiene	688 (65.7)
Medication	865 (82.5)
Household chores	983 (93.8)
Moving around at home	430 (41.0)
Participation outside the home (outdoor activities, hobbies)	783 (74.7)
Help with transportation	940 (89.7)
Co-ordinating care	802 (76.5)
Monitoring (ensuring safety)	812 (77.5)
Co-ordinating in everyday activities	745 (71.1)
Official and financial matters	921 (87.9)
Emotional support	796 (76.0)
Keeping in touch (by phone or computer)	708 (67.6)
Financial support	399 (38.1)
Studying	8 (0.8)
Other care-related issues	67 (6.4)
Intensity of caregiving	1,048 (100.0)
Less than weekly	13 (1.2)
Regularly weekly but not every day	133 (12.7)
Regularly every day (but not at night)	243 (23.2)
Regularly at different times of the day or night	221 (21.1)
Continuously or almost continuously around the clock	431 (41.1)
Non-response	7 (0.7)
Support from family	1,047 (100.0)
No need for support	107 (10.2)
Received reasonable or a lot of support	471 (45.0)
Received only some support	288 (27.5)
Did not receive required support	129 (12.3)
Non-response	52 (5.0)
Support from relatives	1,047 (100.0)
No need for support	350 (33.4)
Received reasonable or a lot of support	129 (12.3)
Received only some support	181 (17.3)
Did not receive required support	296 (28.3)
Non-response	91 (8.7)
Support from friends	1,048 (100.0)
No need for support	312 (29.8)
Received reasonable or a lot of support	123 (11.7)
Received only some support	257 (24.5)
Did not receive required support	264 (25.2)
Non-response	92 (8.8)
Support from support associations for informal caregivers	1,046 (100.0)
No need for support	322 (30.8)
Received reasonable or a lot of support	263 (25.1)
Received only some support	185 (17.7)
Did not receive required support	184 (17.6)
Non-response	92 (8.8)
Support from church social work	1,048 (100.0)
No need for support	630 (60.1)
Received reasonable or a lot of support	36 (3.4)
Received only some support	73 (7.0)
Did not receive required support	176 (16.8)
Non-response	133 (12.7)
Support from public sector social and health care professionals	1,048 (100.0)
No need for support	104 (9.9)
Received reasonable or a lot of support	362 (34.5)
Received only some support	321 (30.6)
Did not receive required support	202 (19.3)
Non-response	59 (5.6)
Use of private paid services	1,048 (100.0)
Needed to pay for private services	273 (26.1)
Did not have to use private services and pay for them	775 (73.9)
Caregiver’s need for social and health services	1,048 (100.0)
Yes	254 (24.2)
No	794 (75.8)
Care duties forcing to leave one’s employment	1,048 (100.0)
Yes	60 (5.7)
No	988 (94.3)

The number of observations varies across variables due to missing responses. Some“non-response” categories reflect missing data that were not consistently captured in the initial tabulation due to spreadsheet formatting

### Factors associated with a weakened economic situation

Table 2 presents the results of logistic regression analysis that investigated factors related to a weakened economic situation among informal caregivers due to providing care for older adults. Model 1 refers to univariate analysis in which each independent variable was included in the model separately, while multivariate analysis adjusted for all the variables is shown in Model 2.

**Table 2:** Association of various caregiving characteristics with negative economic situation

Variables	Model 1^a^ (*n* ≦ 1048)				Model 2^b^ (*n* = 1038)			
	OR	95% CI		*P*-value	OR	95% CI		*P*-value
		LL	UL			LL	UL	
Receipt of informal care allowance (ref. Yes)								
No	1.89	1.42	2.53	< 0.0001*	2.99	1.87	4.78	< 0.0001*
Caregiver age (ref. Older than working age (65 + years old))								
Working age (< 65 years old)	3.83	2.84	5.16	< 0.0001*	3.28	2.06	5.22	< 0.0001*
Care receiver age (ref. 65–79 years old)								
80 + years old	1.18	0.90	1.55	0.226	0.83	0.57	1.20	0.315
Caregiver gender (ref. Female)								
Male	1.15	0.83	1.59	0.406	1.48	0.87	2.49	0.145
Care receiver gender (ref. Male)								
Female	1.68	1.28	2.22	< 0.0001*	1.13	0.71	1.81	0.600
Caregiver’s educational level (ref. Primary education)								
Intermediate education	2.38	1.40	4.04	0.001*	1.23	0.67	2.26	0.509
Higher education	2.90	1.67	5.04	< 0.0001*	0.99	0.51	1.92	0.980
Caregiver’s subjective health (ref. Good or relatively good)								
Moderate	1.20	0.89	1.61	0.245	1.24	0.86	1.79	0.242
Quite poor or poor	2.70	1.81	4.04	< 0.0001*	2.40	1.45	3.97	0.001*
Number of people caring for (ref. 1 person)								
>1 person	1.66	1.08	2.54	0.021*	0.76	0.44	1.31	0.323
Giving care to (ref. Own spouse)								
Other than spouse	2.86	2.15	3.81	< 0.0001*	1.82	1.02	3.24	0.041*
Living in the same household as the care receiver (ref. Yes)								
No	1.99	1.43	2.78	< 0.0001*	0.89	0.47	1.68	0.710
Duration of care (ref. Less than a year)								
1–4 years	1.10	0.62	1.93	0.749	0.95	0.48	1.88	0.886
5–10 years	1.29	0.72	2.30	0.398	0.93	0.45	1.91	0.846
More than 10 years	1.90	1.01	3.58	0.045*	1.35	0.61	2.99	0.452
Non-response	1.07	0.11	10.98	0.952	1.18	0.06	21.98	0.910
Sum of various types of care given	1.10	1.05	1.15	< 0.0001*	1.21	1.12	1.31	< 0.0001*
Intensity of caregiving (ref. Less than weekly)								
Regularly weekly but not every day	0.71	0.22	2.31	0.573	0.23	0.06	0.93	0.039*
Regularly every day (but not at night)	0.56	0.18	1.78	0.325	0.26	0.06	1.05	0.058
Regularly at different times of the day or night	0.61	0.19	1.94	0.402	0.36	0.08	1.53	0.165
Continuously or almost continuously around the clock	0.60	0.19	1.88	0.384	0.31	0.07	1.34	0.117
Non-response	1.20	0.19	7.77	0.848	1.02	0.08	12.80	0.990
Support from family (ref. No need for support)								
Received reasonable or a lot of support	0.69	0.44	1.10	0.117	0.49	0.28	0.86	0.014*
Received only some support	0.87	0.54	1.41	0.568	0.60	0.33	1.07	0.084
Did not receive required support	1.23	0.72	2.12	0.453	0.58	0.29	1.18	0.130
Non-response	0.64	0.30	1.38	0.258	0.71	0.25	2.02	0.514
Support from relatives (ref. No need for support)								
Received reasonable or a lot of support	1.33	0.85	2.08	0.212	1.21	0.68	2.15	0.520
Received only some support	1.34	0.90	2.00	0.151	1.17	0.72	1.92	0.521
Did not receive required support	1.57	1.11	2.21	0.010*	1.11	0.70	1.76	0.647
Non-response	0.42	0.22	0.83	0.012*	0.41	0.14	1.16	0.094
Support from friends (ref. No need for support)								
Received reasonable or a lot of support	0.73	0.44	1.20	0.212	0.94	0.50	1.75	0.839
Received only some support	0.98	0.67	1.42	0.901	0.89	0.56	1.43	0.626
Did not receive required support	1.50	1.05	2.14	0.025*	1.30	0.82	2.07	0.258
Non-response	0.71	0.40	1.24	0.227	1.42	0.55	3.70	0.469
Support from support associations for informal caregiver (ref. No need for support)								
Received reasonable or a lot of support	0.87	0.60	1.25	0.451	0.79	0.48	1.30	0.351
Received only some support	0.89	0.59	1.34	0.574	0.76	0.45	1.28	0.305
Did not receive required support	1.56	1.06	2.29	0.023*	0.96	0.57	1.62	0.877
Non-response	0.46	0.25	0.85	0.013*	0.46	0.17	1.22	0.119
Support from church social work (ref. No need for support)								
Received reasonable or a lot of support	1.89	0.94	3.77	0.073	2.49	1.06	5.86	0.037*
Received only some support	0.97	0.55	1.70	0.914	0.92	0.46	1.87	0.819
Did not receive required support	2.00	1.41	2.85	< 0.0001*	1.79	1.11	2.89	0.018*
Non-response	0.83	0.53	1.29	0.404	2.29	1.01	5.23	0.049*
Support from public sector social and health care professionals (ref. No need for support)								
Received reasonable or a lot of support	1.01	0.59	1.72	0.980	1.04	0.54	2.00	0.906
Received only some support	1.69	1.00	2.86	0.052	1.62	0.86	3.07	0.138
Did not receive required support	2.65	1.54	4.59	< 0.0001*	2.09	1.08	4.04	0.028*
Non-response	0.59	0.24	1.41	0.233	1.58	0.50	5.03	0.437
Use of private paid services (ref. Did not have to use private services and pay for them)								
Needed to pay for private services	1.44	1.07	1.94	0.017*	1.22	0.85	1.76	0.286
Caregiver’s need for social and health services (ref. No)								
Yes	1.62	1.20	2.19	0.002*	1.43	0.97	2.09	0.068
Care duties forcing to leave one’s employment (ref. No)								
Yes	9.96	5.39	18.44	< 0.0001*	7.47	3.50	15.91	< 0.0001*

^a^ Each variable included in the model separately

^b^ All variables included in the model simultaneously

* Statistical significance at significance level of 0.05

When caregiver age was analyzed separately from other variables (Model 1), younger age increased the odds of a weakened economic situation, and the association was statistically significant even after controlling for all variables (Model 2). The age of the care receiver, on the other hand, was not related to the economic situation of the caregiver. The gender of the caregiver did not show a significant association. The female gender of care receivers and the caregiver’s higher educational level (completion of either a bachelor’s or master’s degree) increased the odds of a weaker economic situation. However, mutual adjustment for other independent variables attenuated the association to non-significance. Subjective health was found to be a significant factor in both Model 1 and Model 2, with caregivers’ poorer health significantly associated with their negative economic situation. Similarly, non-receipt of ICA showed a significant association in both univariate and multivariate analyses, and the odds of a weakened economic situation increased when adjusting for other variables (Model 1: 1.89, Model 2: 2.99).

Caring for more than one person and not living in the same household as the care receiver were significantly associated with a weakened economic situation in the univariate analysis. However, when other variables were included, this association was weakened. Providing care to someone other than one’s spouse significantly weakened the economic situation in both univariate and multivariate models. While over 10 years of caregiving increased the odds of a negative economic effect in only univariate analysis, the association of providing various types of care and support remained significant even after adjusting for other variables. In contrast, varying levels of caregiving intensity were not related to the economic situation.

The association between the receipt of support and the caregiver’s economic situation varied depending on the source and level of support. Receiving sufficient support from family members mitigated the negative economic impact of caregiving when other variables were adjusted. Not receiving necessary support from other relatives, friends, and support associations for informal caregivers increased the odds of a weakened economic situation in separate logistic models but not in the full logistic model. In terms of support from the church social work, multivariate analysis revealed that both unmet support needs and receiving reasonable or a lot of support were significantly related to the negative economic effect of caregiving. The absence of required support from the public sector had a significant association in both univariate and multivariate analyses. However, the association between using private paid services and caregivers’ negative economic situation weakened after controlling for other variables.

Using social and health services to cope with caregiving duties increased the odds of a weakened economic situation, but the association became non-significant in the multivariate analysis. Finally, leaving one’s job due to caregiving was identified as the most notable factor associated with the weakened economic situation of the caregiver. Odds ratios remained high even after mutual adjustment for other variables (Model 1: 9.96, Model 2: 7.47).

The findings of the robustness check exhibited consistency across alternative treatments of the “cannot say” category in the dependent variable. In the multinomial logistic regression analyses, the associations for the negative economic outcome relative to the non-negative category were consistent with the main binary model in both direction and magnitude. This finding indicates that the main conclusions were not sensitive to how the “cannot say” responses were handled.

The univariate logistic regression model was analyzed separately based on the gender of caregivers and the results are presented in Supplementary Table 1. Generally, the associations of respective independent variables with the negative economic situation in both male and female caregivers were similar to those in the gender-pooled analyses. However, the tests of interaction terms indicated that the female gender of care receivers (*P* = 0.004), attaining a higher level of education (*P* = 0.010), and poorer subjective health (*P* = 0.041) were more strongly associated with a weakened economic situation in female caregivers compared to their male counterparts.

## Findings from the qualitative study

Inductive content analysis of the responses to the open-ended question regarding the economic impact of caregiving revealed six main themes: dissatisfaction with the informal care allowance, utility of the informal care allowance, increase in care costs, adverse economic consequences, managing costs associated with care, and caregiving at the sacrifice of working life. The sub-themes within each main theme are detailed in Table 3. The subsequent sections elaborate on these main themes using quotes from respondents.

**Table 3:** Six main themes with sub-themes

Sub-themes	Main themes
Viewing the informal care allowance as insufficient	Dissatisfaction with the informal care allowance
The allowance not aligning with the level of care work	
Perceived usefulness of the informal care allowance	Utility of the informal care allowance
The allowance as a significant source of income	
Spending the allowance on care related costs	
Increased/increasing costs due to advancing age and disabilities	Increase in care costs
Cost increases due to changes in the organization of public services	
Extra spending related to caregiving	
Perceiving care services as being expensive	Adverse economic consequences
Insufficient incomes to cover the costs	
Managing care related costs relatively well	Managing costs associated with care
Being prepared for cost increases caused by frailty in old age	
Sharing care related costs with others	
Making efforts to cover the costs	
Not perceiving a major economic impact	
Improved economic situation	
Unable to work in the same capacity as before due to caregiving responsibilities	Caregiving at the sacrifice of working life
Lost income and career prospects due to caregiving	

### Dissatisfaction with the informal care allowance

As previously explained, the Informal Care Allowance (ICA) in Finland includes services for the care receiver, a direct allowance paid to the caregiver for providing care, carer’s leaves, and support services for the caregiver, such as health check-ups and insurance coverage. Although the open-ended question did not specifically mention the ICA system, respondents expressed various opinions about it, particularly regarding the allowance itself. The respondents’ diverse descriptions were categorized into groups, and collating these groups led to two main themes. The first main theme was identified as *dissatisfaction with the informal care allowance*, which included two sub-themes: (1) viewing the informal care allowance as insufficient and (2) the allowance not aligning with the level of care work.

Although the direct allowance paid to the caregiver aims to make informal care possible, the money received does not necessarily ensure this objective. Respondents repeatedly emphasized the insufficient amount of the allowance, which does not cover daily living costs.

*Informal care allowance is not enough for daily expenses. You have to calculate carefully what you buy or need to get.* (No. 291^1^, male, 50–64 years old)

This excerpt effectively illustrates the challenges that informal caregivers face in trying to make ends meet. Caregivers were dissatisfied not only with the amount but also with the allowance being taxable income. Especially caregivers with other income, such as old age pension or salary, expressed concerns about the increased taxation affecting them:

*The informal care allowance is considered taxable income and is taxed together with my pension. After taxes*,* my net monthly income for working round the clock is only a few hundred euros.* (No. 117, female, 65–79 years old)

From the various accounts of respondents, it was also discernible that caregivers perceive the allowance as not corresponding to the amount of care work they are engaged in.

*The amount of work and responsibility never correlates with the allowance.* (No.78, female, 65–79 years old)

The excerpt above aptly describes that the allowance does not align with the level of care work. This perception was another notable feature shaping respondents’ dissatisfaction. The feeling of unfairness was highlighted among respondents who had left employment with a salary before undertaking informal care:

*Even though I receive the highest level of the informal care allowance*,* it is small compared to my previous salary. I feel that caregivers are not valued.* (No. 225, female, 30–49 years old)

### Utility of the informal care allowance

*The utility of the informal care allowance* is another theme identified from the opinions about the ICA system, which presents a contrast with the theme described in the previous section. The perceived utility of the care allowance was as visible as dissatisfaction in the responses. This main theme was comprised of three sub-themes: (1) perceived usefulness of the informal care allowance, (2) the allowance as a significant source of income, and (3) spending the allowance on care related costs. The shared understanding overarching the theme was that the allowance is helpful for household economy regardless of its low level. Even for some caregivers, the allowance was their only and thus a very important source of income. Meanwhile, pensioners tended to regard ICA as a nice addition to their monthly pension and some even showed their gratitude toward the allowance:

*Even a few hundred euros (of the allowance) indeed brings some relief to my small pension.* (No.74, female, 65–79 years old)

The utility of the informal care allowance was also highlighted when caregivers used it to cover care related costs:

*The informal care allowance and our pension currently cover the costs of care.* (No. 113, male, 65–79 years old)

The next excerpt implies that the increasing use of private paid services, as a consequence of insufficient universal public care provision, tends to be covered by the allowance paid to informal caregivers.

*The informal care allowance goes towards the expenses*,* when my husband increasingly needs private paid health services and rehabilitation.* (No. 121, female, 65–79 years old)

It can be argued that the contrast between the two main themes concerning the ICA system, *dissatisfaction with the informal care allowance* and *the utility of the informal care allowance*, is attributable to how informal caregivers understand the meaning and make use of the informal care allowance. Synthesizing various descriptions given by the respondents uncovers a disposition that those who regard the allowance as compensation or remuneration for engaging in informal care work are dissatisfied with the allowance, while others who interpret the allowance more as a token of gratitude or a subsidy to cover the costs of care perceive its usefulness.

### Increase in care costs

*Increase in care costs* was the third main theme identified from various responses. This theme included three sub-themes: (1) increased/increasing costs due to advancing age and disabilities, (2) cost increases due to changes in the organization of public services, and (3) extra spending related to caregiving.

The caregivers were aware of increased and still increasing costs due to the advancing age of care receivers and their deteriorating conditions:

*The progression of sickness over time will have an increasing impact on costs.* (No. 144, female, 65–79 years old)

As the excerpt above demonstrates, the economic impact of cost increases was typically implied by referring to the passage of time and the changes in health status which increase the care needs and thus costs. Furthermore, time has an impact not only on care receivers but also on caregivers. Involvement in caregiving for a long duration has also affected the health and well-being of caregivers, which was reflected in increasing costs:

*My own health has also begun to decline little by little. Of course*,* expenses have increased because now we are both starting to have various ailments!* (No. 17, female, 65–79 years old)

Finland implemented a structural reformation of social and health services in 2023, transferring the responsibility of organizing services from over 300 municipalities to newly established 21 wellbeing services counties (plus the city of Helsinki as one). This organizational transformation is also connected with the cost increases among the respondents:

*The wellbeing county has raised all the user fees*,* so nowadays everything costs more. And the wellbeing county does not grant any discounts on healthcare fees*,* even for persons with low income.* (No.93, female, 50–64 years old)

Costs of care imposed on caregivers and care receivers did not only concern direct care spending such as user fees for health or home care services. Indeed, even more pronounced were various care related expenses that were inevitable for providing care and arranging a better caring environment. These expenses included purchasing foods that meet the special dietary requirements of care receivers, nursing care and hygiene products such as diapers, bed protection, and nitrile gloves. To ease personal care, caregivers often had to buy new clothes suitable for the person being cared for. Increasing medicine costs and dose dispensing fees were also mentioned. Products and services intended to ease caregiving and help informal caregivers, for instance, home renovation, acquisition of assistive devices, and using cleaning services were mentioned too. Especially for those providing distance caregiving, communication costs incurred by mobile phone use and arranging safety devices shaped extra expenses. Finally, transportation costs turned out to be a significant source of extra spending related to care. Fuel expenses strained those who care for their parents living in a remote area or who need to transport care receivers for frequent health checks and medical treatments. Using a taxi was a high cost for those who did not own cars. These additional care related costs were expensive and tended to accumulate, burdening the household economy.

### Adverse economic consequences

Both direct and indirect costs of care negatively impacted the economic situation of caregivers who lived in different informal care contexts: older married couples with pensions, caregivers engaged in long-distance care, and caregivers living in the same household as the person being cared for. This difficult situation, as described by respondents, formed the fourth main theme: *adverse economic consequences*. This theme consisted of two sub-themes: (1) perceiving care services as being expensive, and (2) insufficient incomes to cover the costs.

Whether the received services were from the public sector or private providers, the respondents consistently mentioned that the prices they paid were not inexpensive:

*Private medical doctor visits and examinations*,* which we have to use*,* are expensive.* (No. 352, female, 65–79 years old)

For many respondents, incomes were not enough to cover the costs. Particularly, caregiving and the costs of care were causing economic difficulties for people with low incomes:

*We both have small pensions. Medical expenses for the person I care for are high*,* and we have to dip into our savings to cover regular privately paid physiotherapy.* (No. 131, female, 65–79 years old)

The excerpt indicates that older adults with small pensions are struggling to make ends meet with their present income and have had to resort to their savings if they have any.

### Managing costs associated with care

Despite the increase in care costs and related adverse economic consequences, it is evident from the respondents’ descriptions that they were coping with the challenging situation in various ways. This specific pattern was identified as the fifth main theme, *managing costs associated with care*, which included six sub-themes: (1) managing care related costs relatively well, (2) being prepared for cost increases caused by frailty in old age, (3) sharing care related costs with others, (4) making efforts to cover the costs, (5) not perceiving a major economic impact, and (6) improved economic situation.

*Settling for less does not bring additional costs.* (No. 163, female, 65–79 years old)

This excerpt represents those who were managing care related costs relatively well by adapting to living economically. Meanwhile, others with sufficient pensions did not recognize the adverse effect of care on their household economy, leading to the feeling of managing well:

*Informal caregiving has not substantially affected us because we have both had long careers. As a result*,* both of us receive decent pensions.* (No. 199, male, 80 + years old)

Some respondents were prepared for cost increases caused by old age frailty well in advance, for instance by having private health insurance. Another way to cope with costs incurred by caregiving was sharing daily expenses with other family members, including the person being cared for. The analysis also revealed that some caregivers were making efforts to cover the costs by working two jobs to make ends meet or continuing to work as a freelancer or entrepreneur in retirement.

There were some responses from caregivers who did not perceive a major economic impact. In a few cases, caregivers mentioned an improved economic situation because of receiving the informal care allowance.

### Caregiving at the sacrifice of working life

The open-ended question about the economic impact of caregiving also received many accounts emphasizing that the respondents have been engaged in *caregiving at the sacrifice of their working life*, which was identified to shape the sixth main theme. This theme was comprised of two sub-themes: (1) unable to work in the same capacity as before due to caregiving responsibilities, and (2) lost income and career prospects due to caregiving.

What was repeatedly mentioned concerns the incapacity of working-age caregivers to work as before because of caregiving. Some left their jobs entirely, and others were forced to reduce working hours:

*I had to end my career 4 years too early.* (No. 53, male, 65–79 years old)

The articulation of “had to” in the above excerpt connotes that the work arrangement changes were not voluntary, but the respondent was forced to do so. The next excerpt vividly describes how caregiving is a time-consuming duty that hinders commitment to full-time employment:

*I can work only a few hours a day because I cannot leave the person I cared for alone.* (No. 208, male, 50–64 years old)

For those respondents managing both employed work and care responsibilities, not only a loss of time but also physical and mental strain discouraged them from maintaining their previous level of work commitment and limited their employment options:

*It would be difficult for me to take a full-time job if I were offered such a job. I would not have enough time or energy to provide care for my parents properly and live my own life as well if I had a full-time job. I barely managed with a part-time job last year.* (No. 268, female, 50–64 years old)

Leaving one’s job or reducing working hours due to providing informal care resulted in a loss of income and career opportunities. A spouse carer who was forced to leave her career before the planned retirement age described the lost income, fearing her own low-income retirement:

*I am afraid of the future. I quit my job because I started to take care of my husband. There are still four years before I am eligible to receive the statutory pension. My own pension will be small.* (No. 162, female, 50–64 years old)

In the case of caregivers providing care for their older parents, loss is likely to extend from income and benefits to their future career prospects. The next account suggests that the respondent’s previous paid employment, corresponding to her educational level, and current informal caregiving compensated by the care allowance are mutually exclusive:

*I quit my job and exchanged my salary income*,* equivalent to higher education attainment*,* for my mother’s informal care allowance. Later*,* my father’s care allowance was added.* (No. 226, female, 50–64 years old)

## Interpretation by integrating quantitative and qualitative findings

In this section, we integrate the quantitative and qualitative findings to gain a more nuanced and profound understanding of the research topic. The mixed methods approach with a convergent parallel design merges quantitative and qualitative findings to gain a comprehensive understanding of the research problem [32, 33]. Table 4 displays the integrated results matrix on the factors and their illustration of the negative economic impact of informal caregiving, demonstrating that quantitative and qualitative results support, complement, and enhance each other. The results of the full logistic regression model showed that informal caregivers who were younger, rated their health as poor, cared for someone other than their spouse, provided various types of care, lacked necessary support from the public sector, received adequate support from the church social work or had unmet church social work-related needs, did not receive the ICA, and had to leave their job due to caregiving were identified as being at risk of experiencing a negative economic impact from caregiving.

**Table 4:** Integrated results matrix on the factors of the negative economic impact of informal care giving and their illustration

Quantitative results (factors)	Qualitative results (illustration)
Younger age of caregiver	Unable to work in the same capacity as before due to caregiving responsibilities
	Lost income and career prospects due to caregiving
	Extra spending related to caregiving
Leaving one’s employment due to caregiving	Unable to work in the same capacity as before due to caregiving responsibilities
	Lost income and career prospects due to caregiving
Poor subjective health of the caregiver	Increased/increasing costs due to advancing age and disabilities
Giving care to someone other than one’s spouse	Extra spending related to caregiving
	Unable to work in the same capacity as before due to caregiving responsibilities
	Lost income and career prospects due to caregiving
Engaging in various types of care and support	Perceiving care services as being expensive
	Extra spending related to caregiving
	Unable to work in the same capacity as before due to caregiving responsibilities
	Lost income and career prospects due to caregiving
Lack of necessary support from the public sector	Cost increases due to changes in the organization of public services
	Perceiving care services as being expensive
Receiving adequate support from the church social work or have unmet church social work-related needs	Perceiving care services as being expensive
	Insufficient incomes to cover the costs
Non-receipt of the ICA	Perceived usefulness of the informal care allowance
	The allowance as a significant source of income
	Spending the allowance on care related costs

Caregivers who are younger in age are generally either spouse carers still of working age or adult children caring for their parents. The need for informal caregiving may force spouse caregivers to retire earlier than planned and adult children to either leave their jobs or significantly reduce their working hours [36, 37]. The qualitative analysis shows that the inability to work in the same capacity as before, as well as the loss of income and career prospects experienced by these caregivers, leads to a worsening economic situation [see also 10, 27, 37]. The quantitative analysis further demonstrates that leaving one’s employment due to caregiving has a very strong negative economic impact [e.g., 9]. Additionally, care related expenses such as transportation costs strain the household economy of adult children who are engaged in long-distance care [see 38]. Some studies have also noted that when the person in need of care receives institutional care, the financial burden may be heavy [15, 28]. Moving to institutional care does not usually mean the end of informal care [39].

Caregivers suffering from poor subjective health are typically older spouse carers whose caregiving has lasted for a long period of time. The qualitative analysis indicates that increased and increasing costs due to advancing age and exacerbating health conditions concern both care receivers and caregivers [see also 15, 16, 40].

Both quantitative and qualitative results highlight that caregivers who care for someone other than their spouse face more economic challenges than older married couples with pensions [see also 16]. These caregivers often juggle caregiving responsibilities along with their own family life. Dealing with issues in multiple households results in additional expenses related to caregiving, such as communication and transportation costs, leading to a strained economic situation [e.g., 14, 28]. If the caregivers are adult children who had to leave their jobs or reduce working hours, the negative economic impact of informal care on them is even more pronounced.

Providing various types of care and support, ranging from instrumental support such as household chores and help with transportation, to personal care and hygiene, means that both direct and indirect costs of care are substantial, negatively impacting the economic situation of caregivers. Some of these costs are fully or partially covered by the Finnish welfare state [41], such as home alterations, loan of assistive devices, and purchasing diapers and other care related materials. Additionally, older adults can receive support for transportation to health care services, but this does not extend to distant caregivers’ transportation costs. While there are annual payment ceilings for health care services and medicines, social services fees do not have such. Instead, clients may apply for fee reductions on services like home care, institutional care, and supportive services including cleaning, meals, hygiene, and shopping. These aim to make long-term care more affordable for older adults and may also alleviate the economic burden of caregivers. Irrespective of these public financial supports, our data highlight respondents struggling with the costs of care. This suggests that information on various supportive measures may not reach them or they may not have the time and skills to apply.

The qualitative findings underscore that caregiving is so time-consuming and physically and mentally exhausting that caregivers of working age are not able to continue full-time employment. Leaving their jobs entirely or reducing working hours further exacerbates their economic conditions. There is some evidence that salaried employment of informal caregivers is more likely in countries where social expenditure on formal care is high. However, women and individuals of lower socioeconomic status are less likely to benefit from social expenditures, which results in lower levels of employment [e.g., 10].

Lack of necessary support from the public sector is a significant factor contributing to the negative economic impact, reflecting the transformation of LTC policies under austerity measures that increasingly emphasize individual responsibilities and rely on informal care [42, 43]. The qualitative data reveals that some respondents have no choice but to use expensive private paid services [see 44]. The organizational change in the provision of social and health care services, in connection with the tightening state funding has also led to increased user fees in the public sector, which may deter respondents from using necessary services [see 45]. Furthermore, the finding that caregivers who receive adequate support from the church social work or have unmet church social work-related needs are in adverse economic situations suggests that the church social work serves as a last resort [46]. The qualitative analysis portrays that the caregivers with low incomes, particularly older adults with small pensions, have difficulties making ends meet. This implies that people who cannot afford public and private social and health care services or lack social support are forced to depend on support from the church social work.

The availability of public social and health care services, as well as affordable private paid services, underscores the role of the ICA in the economic situation of informal caregivers. Based on our study sample, in which most respondents received the ICA, informal caregiving negatively affected the economic situation of 28% of respondents and improved that of 12%. These results suggest that the ICA may positively influence caregivers’ household finances by helping to offset caregiving expenses. The qualitative analysis further supports this finding, indicating that even dissatisfied respondents recognize the utility of the care allowance. The multivariate logistic regression model corroborates these findings. The significant association between not receiving the ICA and experiencing a negative economic situation becomes stronger when adjusting for various socio-demographic characteristics and caregiving circumstances. The multivariate model’s results show that the negative economic effect of caregiving without the ICA intensifies while the majority of the univariate model’s significant associations weaken. Thus, the quantitative and qualitative findings suggest that the Finnish cash-for-care scheme could positively impact informal caregivers.

## Discussion and conclusions

This study investigated the subjective views of informal caregivers toward their economic situation in Finland, where the Informal Care Allowance (ICA), a statutory cash-for-care scheme, has been introduced since the early 1990s to support informal care. The survey dataset with both quantitative and qualitative questions enabled the use of a mixed methods approach to address research questions, which involved identifying factors associated with a weakened economic situation of informal caregivers, examining the role of the ICA, and highlighting respondents’ experiences of the economic impact of caregiving.

Our findings highlight that caregivers with low incomes, including older adults with small pensions and working-age caregivers who had to adjust their working arrangements due to care responsibilities, face adverse economic consequences. This is consistent with previous research indicating that informal care is predominantly undertaken by family members in disadvantaged socioeconomic situations, even in Nordic countries with a long tradition of universal public social and health services provision [3, 13, 47]. The results suggest that, similar to other European countries, LTC policies in Finland, influenced by population ageing and austerity measures, are impacting the economic situation of informal caregivers. In Finland, public responsibility for care provision is decreasing, and informal care is increasingly seen as a cost-effective solution. This changing political landscape of long-term care may be reflected in our study sample’s lived experiences regarding the economic impact of caregiving.

Nevertheless, the study’s quantitative and qualitative findings suggest that receiving the ICA could significantly improve conditions for informal caregivers compared to caregiving without it. This suggests that the ICA, Finland’s cash-for-care scheme, is not just a cost-effective policy tool for managing care, but also has the potential to support caregivers and improve the quality of care through cash benefits. The respondents’ mixed feelings about the care allowance suggest that there is still room for the ICA system to achieve its original goal of enhancing informal care for the benefit of care receivers by ensuring adequate social and health services, continuity of caregiving, and support for informal caregivers [19].

Being forced to leave employment due to caregiving constitutes a serious problem not only for the caregivers’ income and career prospects but also for society as a whole in terms of equality. Our findings show that the insufficient level of the informal care allowance negatively affects the income situations of caregivers of working age. This may exacerbate socio-economic differences among those providing informal care and increase inequalities between working-age people with care responsibilities and those without these responsibilities. The observations identified in our study are consistent with a register-based study on contracted informal caregivers (those who receive the ICA) in Finland, which showed that despite receiving the informal care allowance, caregivers experience a decrease in employment and earnings, as well as lower old-age pensions [48]. Additionally, a recent study has found that young contracted informal caregivers aged 20–29 have the highest risk of relying on social assistance after adjusting for all background factors, with this risk decreasing as caregivers age [49]. These findings suggest a growing social divide among citizens in both the short and long term.

As the socio-demographic characteristics and life situations of informal caregivers are diverse, the Finnish cash-for-care scheme, which is basically a “one-size-fits-all” system, does not seem to work for everyone. The qualitative analysis in this study underscored that the respondents interpreted the meaning and utility of the informal care allowance differently. The divergent understandings about the ICA system may be related to the provision in the Act on Support for Informal Care (937/2005) [18], which specifies that the level of the allowance is determined by the binding and demanding nature of the care. The National Audit Office of Finland recently noted that the implementation of the ICA system varies significantly across the wellbeing services counties, including the criteria and benefit levels [50]. The future development of the cash-for-care scheme should take into account the diverse characteristics of informal caregivers and especially support working-age caregivers so that their lost incomes are better compensated by the care allowance or they can reconcile caregiving with employment.

This study has several limitations. First, the survey data may be affected by selection bias. Since the data were obtained from an online survey conducted by a support association for informal caregivers, the results may not fully represent all informal caregivers living in Finland. It is possible that caregivers experiencing economic hardship were more likely to participate in this survey. Additionally, the majority of the respondents in our sample are recipients of the ICA, whereas the nationwide coverage of ICA is only a small portion. This suggests that the economic situations of the remaining informal caregivers, which were not captured in this study, may be even more challenging. The selection bias may also be related to a non-significant factor of caregiver gender, which is inconsistent with the higher economic burden identified in a previous study among male caregivers [30]. Therefore, the findings cannot be generalized, and significant associations and *p*-values identified in logistic regression models should be interpreted with caution.

Second, due to the cross-sectional nature of the data, this study could not establish causality between various factors and the weakened economic situation of informal caregivers. However, the mixed methods approach helped to mitigate this limitation. The open-ended question about how caregiving has influenced participants’ economic situation and respondents’ answers involve implications of the passage of time and the long-lasting nature of care affecting both caregivers and care receivers. Therefore, while the study was cross-sectional in nature, the integration of quantitative and qualitative data from the same participants provided insights that resemble a longitudinal study.

Third, the dependent variable, based on a question about the perceived impact of caregiving on the economic situation, may raise concerns about the lack of objectivity in measuring the outcome. However, research has emphasized the importance of exploring subjective financial assessments, as solely examining income levels may not fully capture lived experiences [51, 52]. Since the study’s goal was to examine how various factors related to caregiving are linked to caregivers’ subjective perceptions of their economic situation, our choice of outcome measurement is justified.

Despite limitations, this study addressed the knowledge gap on economic consequences of informal caregiving under the changing LTC policies and empirically investigated experiences of caregivers. Focusing on Finland, where the policy initiative has gradually shifted the emphasis from public care services to informal care, revealed that the costs of care are already substantially borne by informal caregivers for older adults. The findings of this study call attention to the understanding that the estimated increase in informal care for the next decades will require concrete measures to alleviate economic burdens of caregivers and reduce inequalities within citizens.

## Supplementary Information

Supplementary Material 1.

## Data Availability

The data that support the findings of this study are subject to the following licenses/restrictions: The data are not readily available as they can only be accessed by those who have made an agreement with the proprietor of the data. Requests to access the data should be directed to Carers Finland at https://omaishoitajat.fi/in-english/.

## Abbreviations

LTC: Long-Term Care
ICA: Informal Care Allowance

## Footnotes

1 Respondents (356 individuals) who responded to the open-ended question were assigned their own numbers.
