# Supplementary material for: Economic situation of informal caregivers for older adults in the context of changing long-term care policies: a mixed methods study
Source: BMC Public Health. 2026 Jun 22;26:2797. doi: 10.1186/s12889-026-28179-2 (PMC13637222; doi:10.1186/s12889-026-28179-2)
Supplement: Supplementary file 1 [file 12889_2026_28179_MOESM1_ESM.docx]

**Supplementary Table 1.** Association of various caregiving characteristics with negative economic situation according to caregiver gender^a^

| Variables | **Male** (n≦220) | | | **Female** (n≦828) | | | ***P*-value of interaction**^b^ |
| --- | --- | --- | --- | --- | --- | --- | --- |
|  | OR | 95% CI | | OR | 95% CI | |  |
|  |  | LL | UL |  | LL | UL |  |
| **Receipt of informal care allowance** (ref. Yes) |  |  |  |  |  |  | 0.276 |
| No | 2.83 | 1.36 | 5.89 | 1.82 | 1.32 | 2.51 |  |
| **Caregiver age** (ref. Older than working age (65+ years old)) |  |  |  |  |  |  | 0.519 |
| Working age (<65 years old) | 3.19 | 1.38 | 7.34 | 4.28 | 3.08 | 5.95 |  |
| **Care receiver age** (ref. 65-79 years old) |  |  |  |  |  |  | 0.701 |
| 80+ years old | 1.33 | 0.73 | 2.44 | 1.17 | 0.86 | 1.59 |  |
| **Care receiver gender** (ref. Male) |  |  |  |  |  |  | 0.004* |
| Female | 0.54 | 0.21 | 1.34 | 2.23 | 1.59 | 3.14 |  |
| **Caregiver’s educational level** (ref. Primary education) |  |  |  |  |  |  | 0.010* |
| Intermediate education | 2.14 | 0.96 | 4.76 | 4.84 | 2.23 | 10.51 |  |
| Higher education | 1.10 | 0.44 | 2.73 | 3.24 | 1.52 | 6.91 |  |
| **Caregiver’s subjective health** (ref. Good or relatively good) |  |  |  |  |  |  | 0.041* |
| Moderate | 1.74 | 0.92 | 3.29 | 1.05 | 0.75 | 1.48 |  |
| Quite poor or poor | 1.44 | 0.55 | 3.82 | 3.10 | 1.99 | 4.85 |  |
| **Number of people caring for** (ref. 1 person) |  |  |  |  |  |  | 0.493 |
| >1 person | 2.47 | 0.77 | 7.95 | 1.59 | 1.00 | 2.52 |  |
| **Giving care to** (ref. Own spouse) |  |  |  |  |  |  | 0.642 |
| Other than spouse | 2.56 | 1.27 | 5.18 | 3.07 | 2.23 | 4.23 |  |
| **Living in the same household as the care receiver** (ref. Yes) |  |  |  |  |  |  | 0.239 |
| No | 1.04 | 0.31 | 3.50 | 2.22 | 1.56 | 3.17 |  |
| **Duration of care** (ref. Less than a year) |  |  |  |  |  |  | 0.757 |
| 1-4 years | 0.97 | 0.24 | 3.92 | 1.11 | 0.60 | 2.07 |  |
| 5-10 years | 1.36 | 0.33 | 5.61 | 1.23 | 0.65 | 2.34 |  |
| More than 10 years | 1.33 | 0.29 | 6.04 | 2.07 | 1.03 | 4.15 |  |
| Non-response | n/a | n/a | n/a | 1.11 | 0.11 | 11.48 |  |
| **Sum of various types of care given** | 1.05 | 0.95 | 1.17 | 1.11 | 1.05 | 1.17 | 0.349 |
| **Intensity of caregiving** (ref. Less than weekly) |  |  |  |  |  |  | 0.367 |
| Regularly weekly but not every day | n/a | n/a | n/a | 0.72 | 0.22 | 2.36 |  |
| Regularly every day (but not at night) | 0.78 | 0.23 | 2.64 | 0.59 | 0.19 | 1.88 |  |
| Regularly at different times of the day or night | 0.49 | 0.20 | 1.21 | 0.60 | 0.19 | 1.93 |  |
| Continuously or almost continuously around the clock | 0.78 | 0.38 | 1.61 | 0.53 | 0.17 | 1.66 |  |
| Non-response | n/a | n/a | n/a | 1.60 | 0.23 | 11.27 |  |
| **Support from family** (ref. No need for support) |  |  |  |  |  |  | 0.299 |
| Received reasonable or a lot of support | 0.86 | 0.33 | 2.23 | 0.64 | 0.38 | 1.09 |  |
| Received only some support | 1.26 | 0.48 | 3.28 | 0.77 | 0.44 | 1.34 |  |
| Did not receive required support | 3.10 | 0.99 | 9.74 | 0.96 | 0.51 | 1.79 |  |
| Non-response | 0.44 | 0.08 | 2.40 | 0.71 | 0.30 | 1.69 |  |
| **Support from relatives** (ref. No need for support) |  |  |  |  |  |  | 0.450 |
| Received reasonable or a lot of support | 1.28 | 0.49 | 3.38 | 1.34 | 0.81 | 2.22 |  |
| Received only some support | 2.25 | 0.96 | 5.29 | 1.17 | 0.74 | 1.85 |  |
| Did not receive required support | 2.38 | 1.13 | 5.02 | 1.41 | 0.96 | 2.08 |  |
| Non-response | 0.29 | 0.06 | 1.36 | 0.47 | 0.22 | 1.00 |  |
| **Support from friends** (ref. No need for support) |  |  |  |  |  |  | 0.499 |
| Received reasonable or a lot of support | 0.32 | 0.07 | 1.53 | 0.81 | 0.48 | 1.39 |  |
| Received only some support | 1.29 | 0.59 | 2.81 | 0.90 | 0.59 | 1.38 |  |
| Did not receive required support | 2.14 | 0.98 | 4.65 | 1.38 | 0.92 | 2.05 |  |
| Non-response | 0.74 | 0.26 | 2.13 | 0.69 | 0.35 | 1.35 |  |
| **Support from support associations for informal caregiver** (ref. No need for support) |  |  |  |  |  |  | 0.995 |
| Received reasonable or a lot of support | 0.74 | 0.32 | 1.68 | 0.90 | 0.60 | 1.37 |  |
| Received only some support | 0.88 | 0.34 | 2.25 | 0.90 | 0.57 | 1.41 |  |
| Did not receive required support | 1.49 | 0.67 | 3.32 | 1.56 | 1.01 | 2.43 |  |
| Non-response | 0.43 | 0.14 | 1.29 | 0.45 | 0.21 | 0.96 |  |
| **Support from church social work** (ref. No need for support) |  |  |  |  |  |  | 0.979 |
| Received reasonable or a lot of support | 2.52 | 0.48 | 13.10 | 1.78 | 0.83 | 3.85 |  |
| Received only some support | 0.97 | 0.32 | 2.93 | 0.95 | 0.50 | 1.83 |  |
| Did not receive required support | 1.83 | 0.86 | 3.91 | 2.04 | 1.37 | 3.03 |  |
| Non-response | 0.69 | 0.30 | 1.59 | 0.87 | 0.51 | 1.48 |  |
| **Support from public sector social and health care professionals** (ref. No need for support) |  |  |  |  |  |  | 0.712 |
| Received reasonable or a lot of support | 0.77 | 0.19 | 3.13 | 1.06 | 0.59 | 1.89 |  |
| Received only some support | 1.74 | 0.43 | 7.05 | 1.62 | 0.91 | 2.87 |  |
| Did not receive required support | 3.00 | 0.70 | 12.83 | 2.52 | 1.39 | 4.57 |  |
| Non-response | n/a | n/a | n/a | 0.73 | 0.29 | 1.82 |  |
| **Use of private paid services** (ref. Did not have to use private services and pay for them) |  |  |  |  |  |  | 0.732 |
| Needed to pay for private services | 1.58 | 0.85 | 2.94 | 1.39 | 0.99 | 1.96 |  |
| **Caregiver’s need for social and health services** (ref. No) |  |  |  |  |  |  | 0.119 |
| Yes | 2.66 | 1.34 | 5.26 | 1.45 | 1.03 | 2.04 |  |
| **Care duties forcing to leave one’s employment** (ref. No) |  |  |  |  |  |  | 0.464 |
| Yes | 6.70 | 2.02 | 22.22 | 11.30 | 5.50 | 23.19 |  |

Note: ^a^ Each variable included in the model separately. ^b^ Test of interaction term shows that the gender of caregiver moderates the association between the independent variable and negative economic situation if the *P*-value is significant.
